# Localized *Histoplasma capsulatum* osteomyelitis of the fibula in an immunocompetent teenage boy: a case report

**DOI:** 10.1186/1471-2334-13-132

**Published:** 2013-03-11

**Authors:** Lu Huang, Yongping Wu, Xudong Miao

**Affiliations:** 1 Department of Orthopedics, the Second Affiliated Hospital, School of Medicine, Zhejiang University, 88 Jie Fang Road, Hangzhou, Zhejiang 310009, P.R. China

**Keywords:** *Histoplasma capsulatum*, Histoplasmosis, Immunocompetence, Osteomyelitis

## Abstract

**Background:**

Infection of local bone with *Histoplasma capsulatum* is rare and difficult to diagnosis, and occurs particularly in immunocompetent subjects, who are more likely to be affected by a wide range of organisms.

**Case presentation:**

An 11-year-old boy presented with localized histoplasmosis osteomyelitis in the left fibula without any evidence of abnormal immunological function or systemic disease. After surgical clearance of the lesion and homologous cancellous bone, the patient was treated orally with voriconazole for 6 months. The patient completely recovered with full function of his left leg during the 5-year follow-up.

**Conclusions:**

Histoplasmosis osteomyelitis can occasionally occur in immunocompetent individuals and can be complete cured by surgical clearance of the lesion and antibiotic treatment.

## Background

*Histoplasma capsulatum*, a thermally dimorphic fungus, is endemic across North and Central America, particularly in the region of the Mississippi river. Exposure to the organism can cause fatal disseminated fungemia in immunocompromised patients, but only results in an asymptomatic infection in most immunocompetent subjects [1]. Although the organism is found worldwide, cases of histoplasmosis are rare in China. In the past several decades, only a few cases have been reported, and infection with *H. capsulatum* only in bone is rare. In addition, infection with *H. capsulatum* is often neglected by Chinese physicians when they differentially diagnose opportunistic infections in patients with abnormal immunological functions [2-10].

We diagnosed and treated one patient, who had no sign of abnormal immune function or other chronic disease, but with histoplasmosis osteomyelitis only in the fibula. We followed the patient for 5 years, and he did not have any adverse consequences. We report this case to discuss our experience in the diagnosis and treatment of patients with histoplasmosis osteomyelitis of the bone.

## Case presentation

An 11-year-old boy was admitted to our hospital on February 20, 2006. He complained of having swelling and pain of the lateral portion of the lower leg for 1 week, accompanied by local erythema. He had no fever or chills at the time of admission. In general, he was healthy and had no systemic chronic disease, nor a history of injury in the lower extremity. He denied recent travel or drug use. He was a native resident. The patient denied exposure to any infectious patients and any history of infusion.

Physical examination revealed a body temperature of 37°C, blood pressure of 118/78 mmHg, heart rate of 78 beats/min, respiratory rate of 19 breaths/min, and body weight of 46 kg. The patient appeared to be in good nutritional condition. There was no lymphadenopathy or hepatomegaly noted, and findings in his heart and lungs were unremarkable. There was a 2 × 1 cm red skin nodule with mild tenderness in the lateral malleolus near the left ankle of the fibula. The movement of his ankle joint was satisfactory. His arterial pulse was detected on the dorsum of foot, and he was able to move his toe normally.

Laboratory testing indicated a blood white cell count of 8.6 × 10^9^/L, with neutrophils accounting for 58.3% and lymphocytes for 31.6%. The hematocrit was 36.0%. He was negative for anti-human immunodeficiency virus, anti-syphilis, and anti-hepatitis C virus antibodies, anti-hepatitis A virus immunoglobulin, and hepatitis B surface antigen. The concentration of serum alkaline phosphatase was 226 U/L, slightly higher than the cutoff for abnormal values: >140 U/L), and total bilirubin was 0.19 mg/dL, which was lower than the cutoff value of 0.30 mg/dL. The concentrations of plasma electrolytes, creatinine, urea nitrogen, and albumin were within normal ranges.

**Figure 1:**
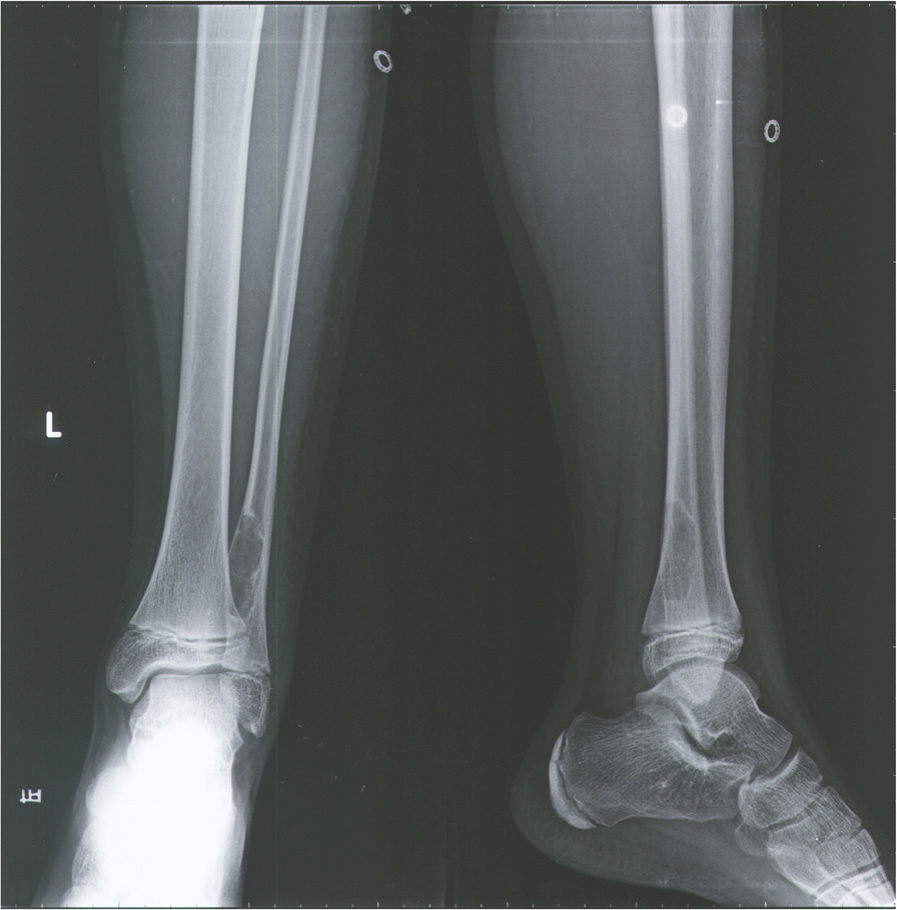
X-ray imaging of the left ankle revealed a cystic transparent lesion and absorption of the inner edge of bone at the distal fibula.

**Figure 2:**
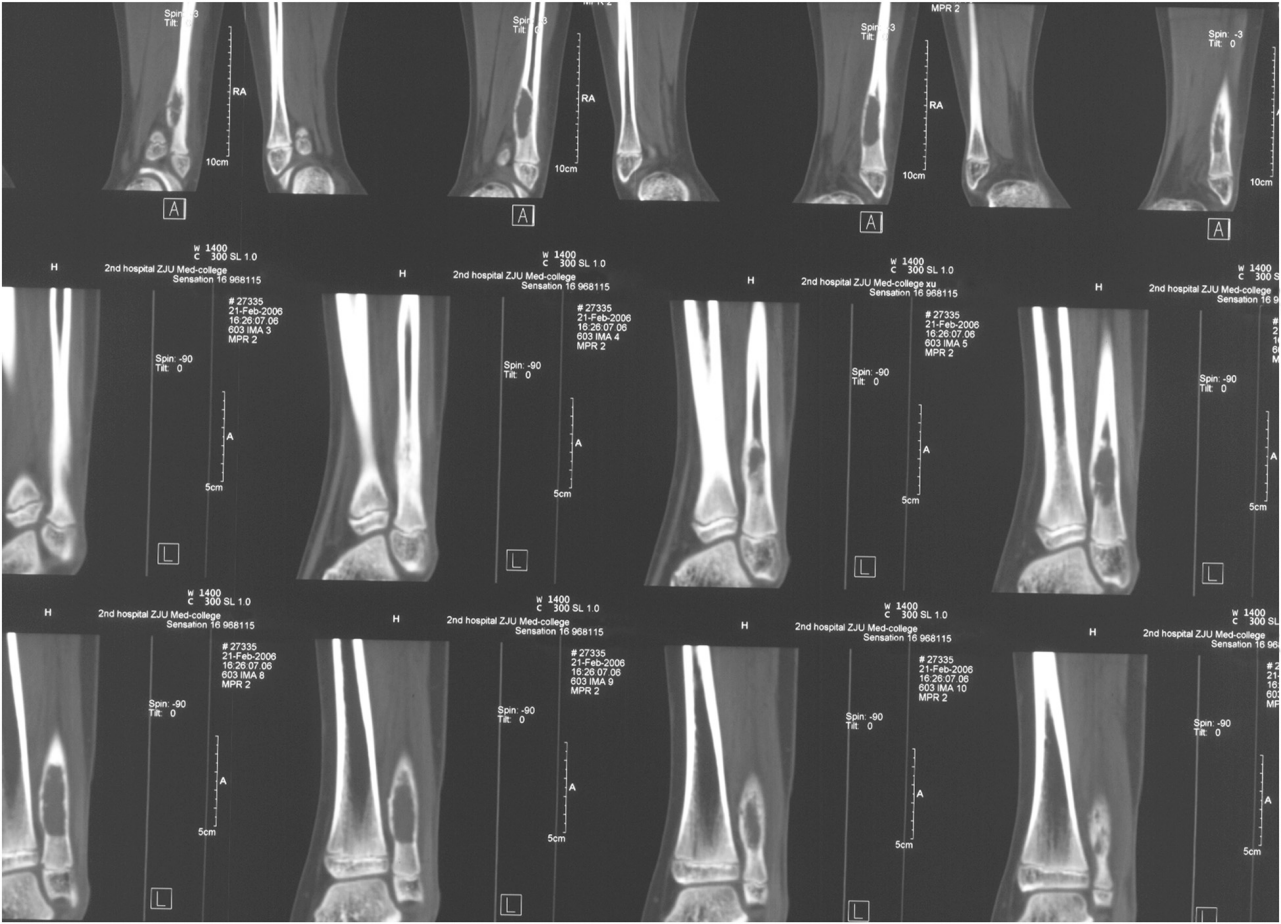
Computes tomography of the distal left fibula showing a cystic and low density image with eccentric enlargement as well as discontinuous bone vortex and decreased cortical thickness.

**Figure 3:**
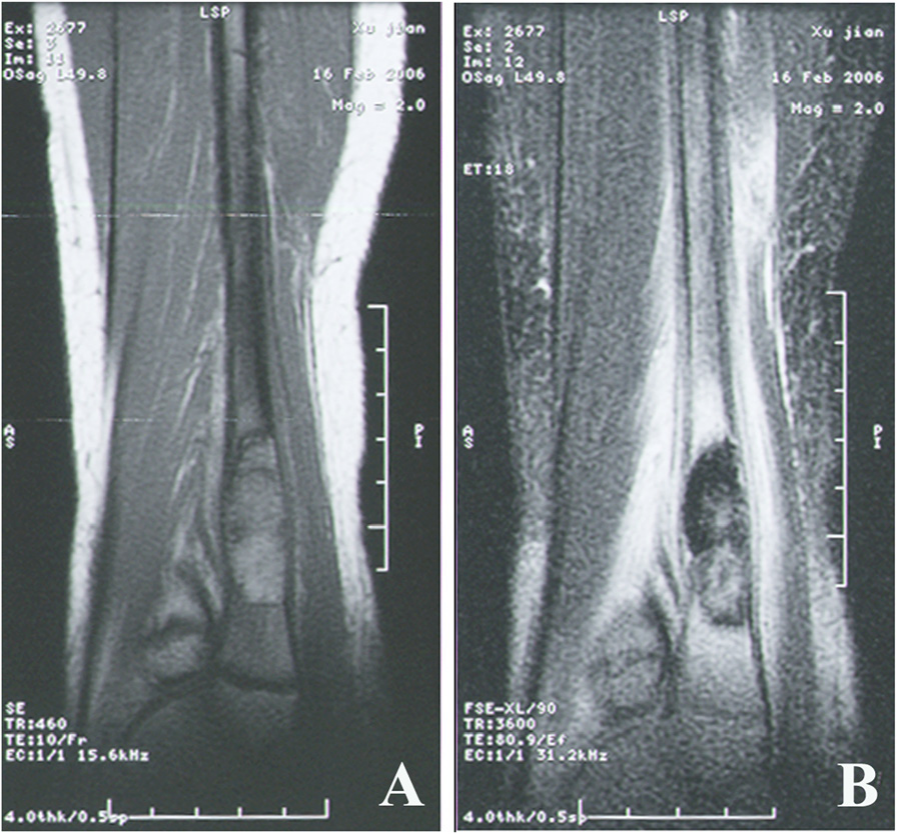
**Magnetic resonance imaging (MRI). A: **MRI revealed a slightly increased size of the lower left fibula, a low signal of T1WI with uneven inner signal. **B: **MRI also showed a medium or low T2W1 signal of the lower fibula and the surrounding soft tissues with high signal and unclear edges.

**Figure 4:**
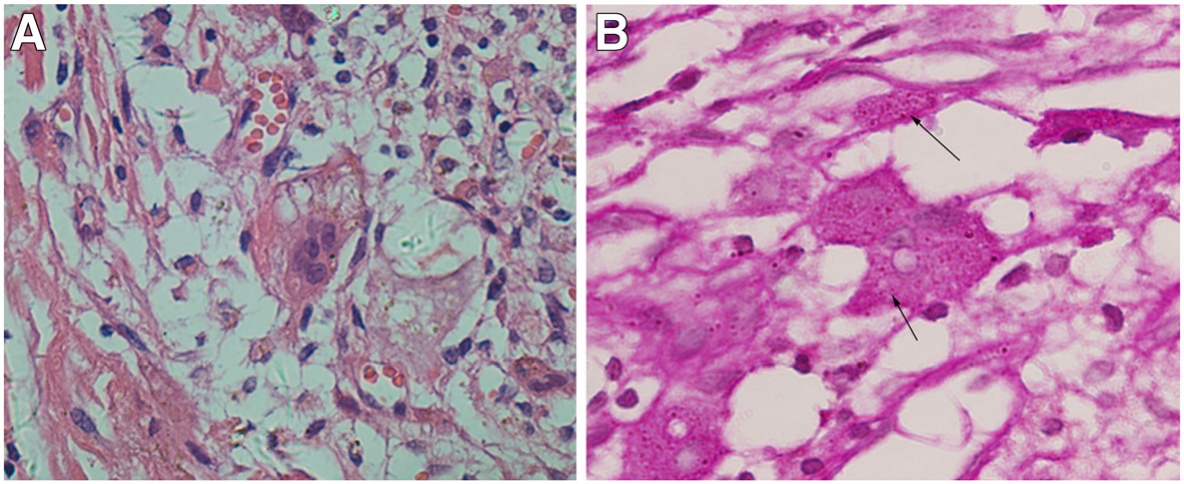
***Histoplasma capsulatum*. A: ***H. capsulatum *can be observed in the plasma of histocytes and multinuclear giant cells and there were clear haloes around the bacteria. Hematoxylin & eosin x400. **B:***H. capsulatum *can be clearly demonstrated in red by periodic acid-Schiff (PAS) stain (arrows): the shapes of the spores are round or oval, mostly of identical appearance. There are empty halos around the bacteria. PAS x400.

**Figure 5:**
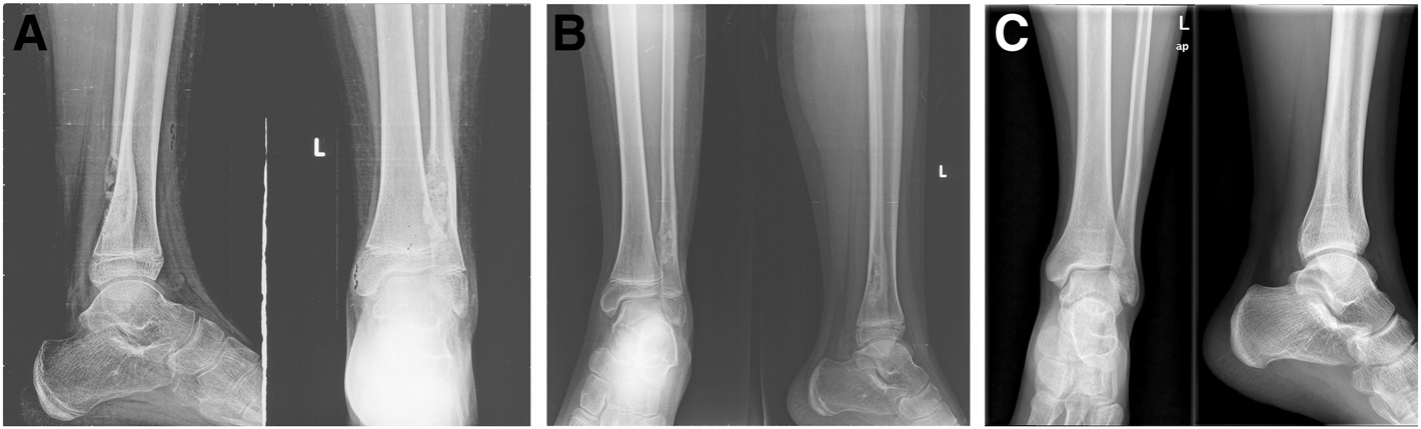
**X-ray imaging of the distal fibula. A: **Three months after the surgery. **B: **Six months after the surgery. **C: **Five years after the surgery.

Radiological imaging revealed a cystic lesion with bone growth, absorption, and diffused edges in his left ankle (Figure 1). Computed tomography (CT) of the lower extremities displayed possible bone cysts or osteomyelitis in the distal left fibula, accompanied by a discontinuous bone cortex and decreased cortical thickness, but without surrounding soft tissue swelling (Figure 2). Magnetic resonance imaging (MRI) of the lower extremities found a slightly increased size of the distal left fibula with a low signal of uneven T1WI and T2WI. MRI also showed extensive low T1W1 and high T2W1 in the surrounding soft tissues with unclear edges (Figure 3). The patient was suspected of having a bone tumor or infectious cyst. Two days after admission, the patient underwent surgery on the distal lesion in the left fibula. During the surgery, we observed that the lateral cortical bone was still intact and that the subcortex was filled with brown fleshy tissue 3 × 1 × 1 cm in size. Intraoperative examination of a frozen section suggested nonossifying fibroma. The lesion tissues and narrow margin were scraped using a curette, and the tissue cavity was treated sequentially with carbolic acid, alcohol, and hydrogen peroxide. Finally, the cavity was filled with homologous cancellous bone and the wounds were sutured. Histological analysis revealed round and oval spores as well as granulomatous inflammatory cells in the lesion tissue sections, accompanied by positive periodic acid-Schiff staining and periodic acid methenamine-silver staining, but a negative smear for acid-fast staining, indicating *H. capsulatum* (Figure 4). The patient was diagnosed with a localized histoplasmosis osteomyelitis and was treated orally with voriconazole (400 mg once daily) for 6 months. There was no significant discomfort or adverse effects during the anti-fungal treatment. The patient was in a stable condition without fever after the surgery, and his wound healed well. The patient was discharged with outpatient instructions to avoid weight-bearing for 1 week after the surgery. The patient was followed up for 5 years (Figure 5). He regained normal function in his left leg without any adverse complaints.

## Conclusions

*H. capsulatum* infection causes histoplasmosis, which usually occurs in the lungs. The environmental reservoir of *H. capsulatum* is soil [11]. People acquire *H. capsulatum* infection usually through the inhalation of conidial forms of the organism present in the environment, such as in soils exposed to chickens. Our case had no obvious source of the infection. He denied a recent history of travel and was living in the countryside in Zhejiang Province, Southeast China, where there are many breeding chickens and ducks in the farmhouses. His father was a chicken farmer and chickens entered his home. He remembered that he used to clean the chicken coop to help his family’s poultry business before he became ill. It is possible that he acquired the *H. capsulatum* infection from the poultry. Indeed, the prevalence of potential *H. capsulatum* infection in Southeast China is higher than that in Northwest China [9]*.*

Infection of immunocompetent subjects with *H. capsulatum* usually causes either asymptomatic or mild influenza-like illnesses with fever, headache, malaise, cough, and chest pain, which spontaneously disappear within a few days. Infected individuals can carry *H. capsulatum* for many years. When the patient becomes immunosuppressed he can develop disseminated histoplasmosis that can affect the lungs, central nervous system, liver, spleen, and rheumatologic, ocular, and hematologic systems [12]. However, patients with histoplasmosis in the bone and muscle tissues are extremely rare [12]. There are 25 reported cases of confirmed muscle-bone histoplasmosis. Among them, most patients had disseminated histoplasmosis and there were only six patients who suffered from simple bone histoplasmosis, with a lesion in the distal radius or carpal bones [13-17]. There was only one reported case with lower limb histoplasmosis. A patient with non-Hodgkin's lymphoma also had *H. capsulatum* infection in the tibia [14]. Therefore, to the best of our knowledge, this is the first report of a localized histoplasmosis osteomyelitis in the fibula in an immunocompetent young boy.

Rather than filling the surgical lesion with bone cement or antibiotic beads as in adult patients, the surgical cavity of the patient was filled with homologous cancellous bone to reconstruct a stable distal tibiofibular syndesmosis and ankle joint. Indeed, radiological images and physical examination have demonstrated that the function and structure of the surgical muscles and bone tissues completely recovered. The patient was able to take part in normal and strenuous exercise, and even soccer. Itraconazole and amphotericin B were listed as the drugs for the treatment of histoplasmosis [18]. Amphotericin B has been accepted as a standard drug for the treatment of patients with severe illness, but it occasionally has severe side-effects. Amphotericin B has been recommended for induction therapy for disseminated histoplasmosis in patients with acquired immunodeficiency syndrome, and either amphotericin B or an oral azole antifungal agent (itraconazole) for maintenance. Conazoles can be used as an alternative for the treatment of disseminated histoplasmosis, and itraconazole may be used for both induction and maintenance treatment [19]. However, according to itraconazole labeling, there is not enough evidence for its use in children. Consequently, we treated the 11-year-old boy with voriconazole, a conazole with clear labeling and guidance for its use in teenagers. The prognosis was very satisfactory, as the follow-up results indicated.

In summary, we report a unique case of localized histoplasmosis osteomyelitis without evidence of immunocompromise. This disease was difficult to diagnose using common radiological examinations. We found that surgical removal of the lesion and local treatment with autologous bone transplantation, together with systemic treatment with antibiotics were effective for the control of infection and functional recovery.

### Consent

Written informed consent was obtained from the patient for publication of this case report and any accompanying images. A copy of the written consent (in Chinese) is available for review upon requested.

## Competing interests

The authors declare that they have no competing interests.

## Authors’ contributions

LH participated in the sequence alignment and drafted the manuscript. YW participated in critical revision of the article. XM was in charge of the diagnosis and treatment of the case. All authors read and approved the final manuscript.

## Pre-publication history

The pre-publication history for this paper can be accessed here:

http://www.biomedcentral.com/1471-2334/13/132/prepub
